# lncRNA LINC00355 Acts as a Novel Biomarker and Promotes Glioma Biological Activities via the Regulation of miR-1225/FNDC3B

**DOI:** 10.1155/2021/1683129

**Published:** 2021-09-24

**Authors:** Zhen-yong Qi, Li-li Wang, Xu-liang Qu

**Affiliations:** ^1^Department of Laboratory Medicine, The Shouguang People's Hospital, Weifang, Shandong Province, China; ^2^Department of Laboratory Medicine, The Qingzhou City & Child Healthcare Hospital, Qingzhou, Shandong Province, China; ^3^Department of Laboratory Medicine, The Laizhou People's Hospital, Laizhou, Shandong Province, China

## Abstract

**Background:**

Accumulating evidence has implicated long noncoding RNAs (lncRNAs) in glioma progression. Here, we aimed to explore the potential roles of a novel lncRNA, LINC00355, in glioma and to clarify the underlying mechanisms.

**Methods:**

RT-PCR was used to examine the relative expressions of LINC00355 in glioma cell lines and specimen samples. The clinicopathological and prognostic significances of LINC00355 in glioma patients were statistically analyzed. To determine cell activities, CCK-8, clonogenic assays, flow cytometry, migration, and invasion assays were performed. Moreover, the potential mechanisms of LINC00355 were investigated by bioinformatics assays and luciferase reporter assays.

**Results:**

LINC00355 expression was increased in glioma cell lines and specimens, and higher LINC00355 expression predicted advanced clinical progress and reduced overall survival and disease-free survival in glioma patients. Functionally, LINC00355 depletion promoted cell proliferation, invasion, and migration in glioma cells and induced apoptosis of glioma cells, whereas LINC00355 upregulation resulted in the opposite effects in vitro. Mechanistic assays revealed that LINC00355 as a sponge for miR-1225 repressed fibronectin type III domain-containing 3B (FNDC3B) expressions.

**Conclusion:**

Our findings revealed the tumor-promotive roles of LINC00355 in the progression of glioma, indicating that LINC00355 exhibited ceRNA functions via modulating miR-1225/FNDC3B axis.

## 1. Introduction

Glioma represents the most prevalent and fatal brain neoplasm in both men and women, accounting for >75% of primary intracranial brain tumors [1]. Compared with all the other glioma types, glioblastoma (GBM) has a distinctly higher incidence, with an unfavorable long-term survival of < eight months, due to its reinforced metastasis and heterogeneous cytogenetic and molecular metamorphosis [2, 3]. Moreover, because of the sluggish development in exploring the potential pathogenesis, the effective therapeutic strategies for this tumor are limited [4]. Thus, it is of significant scientific and clinical value to explore the potential mechanism involved in tumor metastasis for the further determination of sensitive clinical biomarkers and potential therapeutic targets.

Noncoding RNAs are considered to have the limited protein-coding ability, such as tRNA, snoRNA, miRNAs, and some other types of RNAs [5]. Long noncoding RNAs (lncRNAs) are a newly emerged class of noncoding RNAs that contain greater than 200 nucleotides and have been evidently demonstrated to be extensively transcribed in the genomes [6, 7]. For a long time, lncRNAs are believed to be “transcriptional noise.” However, more and more evidences in recent years suggest lncRNAs as essential regulators in DNA replication, transcription, and gene expressions [8, 9]. In cellular function, lncRNAs are confirmed to play an influential role in numerous biological processes, including pluripotency of stem cells, cellular differentiation, cell replication, and programmed cell death [10]. The regulatory functions of lncRNAs in tumor initiation and progression via the modulation of tumor-promotive and antioncogenic pathways have attracted growing attention [11, 12]. Many tumor-associated lncRNAs have been functionally characterized [13, 14]. However, generous lncRNAs have not been functionally characterized so far and the potential mechanisms underlying their tumor-related functions on the progression and metastasis of glioma remained largely unclear.

MicroRNAs (miRNAs) are a class of small noncoding RNAs, ∼18–25 nucleotides, which display abnormal expressions in various cancers and serve as tumor suppressors or tumor promoters via inhibiting translation of tumor-related genes [15, 16]. An emerging hypothesis, known as the competing endogenous RNA (ceRNA), reveals that some special lncRNAs could “talk” with mRNAs of they have the same miRNA binding sites [17]. The cross-modulation between lncRNAs and miRNAs highlights their imperative effects on the development and metastasis of glioma, promoting our group to further elucidate the underlying mechanisms.

In this study, for the first time, we identified a novel glioma-associated lncRNA, lncRNA LINC00355 (LINC00355), which was demonstrated to be overexpressed in glioma specimens and predict a poor clinical prognosis of glioma patients. Then, we performed loss-of-function and gain-of-function assays, confirming LINC00355 as a tumor promoter in glioma. Mechanistic studies identified a novel regulatory mechanism of LINC00355/miR-1225/FNDC3B axis in carcinogenesis and metastasis, suggesting a novel clue for tumor treatments.

## 2. Methods

### 2.1. Tissue Sample Collection

Specimens from glioma and matched normal adjacent tissues were collected from 121 patients at the Laizhou People's Hospital from March 2013 to June 2016 with written informed consents and approvals from the Ethics Committee of the Laizhou People's Hospital. Before surgery resection, the patients received no pre- or postoperative adjuvant therapy. The tissue samples were snap-frozen and preserved at -80°C.

### 2.2. Cell Culture and Transfection

Cells including NHAs (as control cells) and glioma cells (T98G, LN229, LN18, A172, and U251) were obtained from TongHe Biology (Hangzhou, China). They were cultured using RPMI-1640 media in an incubator. Cell transfection was conducted using Lipofectamine 2000 transfection kits (LumingBio, Dalian, Liaoning, China). The miR-1225 mimics, NC mimics (negative control), miR-1225 inhibitors, NC inhibitors (negative control), and siRNAs (siRNAs target LINC00355: si-LINC00355#1, si-LINC00355#2, and si-LINC00355#3; siRNAs target FNDC3B: si-FNDC3B#1, si-FNDC3B#2, and si-FNDC3B#3) were obtained from YunRun Biological Company (Nantong, Jiangsu, China). The LINC00355 sequence was constructed into pcDNA3.1 empty vector to overexpress LINC00355 (ov-LINC00355), and the construction was conducted by NaYe Biology (Hangzhou, China).

### 2.3. Real-Time PCR

Total RNAs and miRNAs were separately extracted using TRIzol kits (YiRanBio, Changsha, Hunan, China) and QIAGEN miRNeasy kits (JianlunBio, Guangzhou, Guangdong, China), respectively. For FNDC3B mRNA and LINC00355 detection, we firstly used a Transgen cDNA Synthesis SuperMix kits (SanjianBio, Binhai, Tianjin, China) to transcribe mRNA into cDNA. Then, real-time PCR reactions were conducted by employing qPCR Master kits (GuangjingBio, Suzhou, Jiangsu, China). For miR-1225 examination, one-step miRNA qPCR kits (Genetimes, Taicang, Jiangsu, China) were used. GAPDH was used as internal controls of mRNA and lncRNA detection, and U6 was used as miRNA examination. Relative mRNA, lncRNA, or miRNA expression was calculated using the 2^−ΔΔCt^ method. Sequences of relevant primers are displayed in Table 1.

### 2.4. Cell Viability Detection

Cells after designated treatment were placed into plates (96-well; at a density of 3.5 × 10^3^ cells/well) and kept in an incubator (37°C, 5% CO_2_) for indicated time periods. Afterwards, Dojindo CCK-8 reagents (15 *μ*l/well; BioDe, Wuhan, Hubei, China) were placed into the plates. After incubation for 2 h, a microplate reader was applied to detect the absorbance values at 450 nm.

### 2.5. Clonogenic Assay

The treated cells were placed into plates (6-well; 500 cells) and cultured for 16 days. Until the cell colonies were visible under the naked eyes, paraformaldehyde was used to fix the colonies and crystal violet was employed to treat the colonies, followed by being imaged using a microscope.

### 2.6. Cellular Apoptosis Examination

The treated cells were trypsinized, harvested, and resuspended in PBS buffer. Thereafter, the cells were stained with propidium iodide (6 *μ*g/*μ*l; BoanBio, Hangzhou, Zhejiang, China) and Annexin V-FITC (15 *μ*g/*μ*l; Biogene, Guangzhou, Guangdong, China). The cells were kept away from light for 20 min, washed using PBS, and then measured by flow cytometry machine.

### 2.7. Caspase 3/9 Activity Detection

We used Abcam caspase 3/9 activity detection kits (JissKangBio, Qingdao, Shandong, China) to determine the caspase 3/9 activities of tumor cells after treatment with LINC00355 siRNAs or ov-LINC00355 plasmids. In short, the supernatants of the cell lysates were prepared using the lysis buffer and centrifuging. Subsequently, the supernatants were added with 2× reaction buffer, followed by adding DTT and DEVD-pNA reagents (5 *μ*l; 4 mM). After incubation for 100 min, the absorbance values at 405 nm were measured output.

### 2.8. Wound Healing Assay

The cells (3.5 × 10 [5]) after treatments were trypsinized and replaced into plates (24-well). The cells were cultured for 24 h (the cell confluent reached 100%). Subsequently, the cellular monolayers were scratched by the use of a plastic pipette tip (200 *μ*l) and the artificial wounds were generated. The wound closures were photographed using a microscope at 0 h and 48 h after the wounds were created.

### 2.9. Transwell Assay

The treated cells (1.5 × 10^5^ in 200 *μ*l) were trypsinized and placed into the Costar Transwell upper chambers which were treated with Matrigel. The lower chambers were then supplemented with 650 *μ*l media containing 15% serum. The plates were kept in an incubator (37°C, 5% CO_2_) for 36 h, and paraformaldehyde and crystal violet were employed to treat the cells on the lower sides. After washing with PBS, a microscope was utilized to take pictures of the cells.

### 2.10. Subcellular Fractionation Assay

Briefly, we first isolated the nuclear and cytoplasmic extracts from U251 cells using Thermo Fisher PARIS kits (Guanghe, Ningbo, Zhejiang, China) according to the kits' protocols. Afterwards, the RNAs were, respectively, isolated from nuclear and cytoplasmic extracts. Finally, U6, GAPDH, and LINC00355 were evaluated by RT-PCR. U6 was used as nuclear control, and the cytoplasm control was GAPDH.

### 2.11. Luciferase Activity Reporter Assay

The predicted miR-1225 binding site in the 3′-UTR of FNDC3B mRNA sequence was constructed into pGL3 luciferase reporter vectors (wild-type: WT), and corresponding mutant binding site sequence was also constructed (mutant: MUT). The reporters were named as follows: FNDC3B WT and FNDC3B MUT. Correspondingly, the wild-type or mutant-type LINC00355 sequence was, respectively, constructed into pGL3 empty vectors to form LINC00355 WT or LINC00355 MUT luciferase reporters. The vectors were constructed by NaYe Biological Company (Hangzhou, Zhejiang, China). After the cells were attached on the plates, they were cotransfected with designated luciferase reporter vectors and miR-1225 mimics or controls. After 48 h, the luciferase activities of glioma cells were determined by the use of Promega luciferase reporter detection kits (JunShangBio, Chengdu, Sichuan, China).

### 2.12. Statistical Analysis

Data were analyzed by the SPSS statistics software (SPSS Inc., Chicago, IL, USA). Differences between two groups were assessed using Student's *t*-test or chi-square test. Multiple groups were compared with a one-way ANOVA. To estimate overall survival (OS) and disease-free survival (DFS), Kaplan-Meier survival analysis was carried out. The survival data were analyzed by the use of univariate and multivariate analyses. *p* < 0.05 was considered statistically significant.

## 3. Results

### 3.1. LINC00355 Was Upregulated in Glioma and Associated with Poor Prognosis

**Figure 1:**
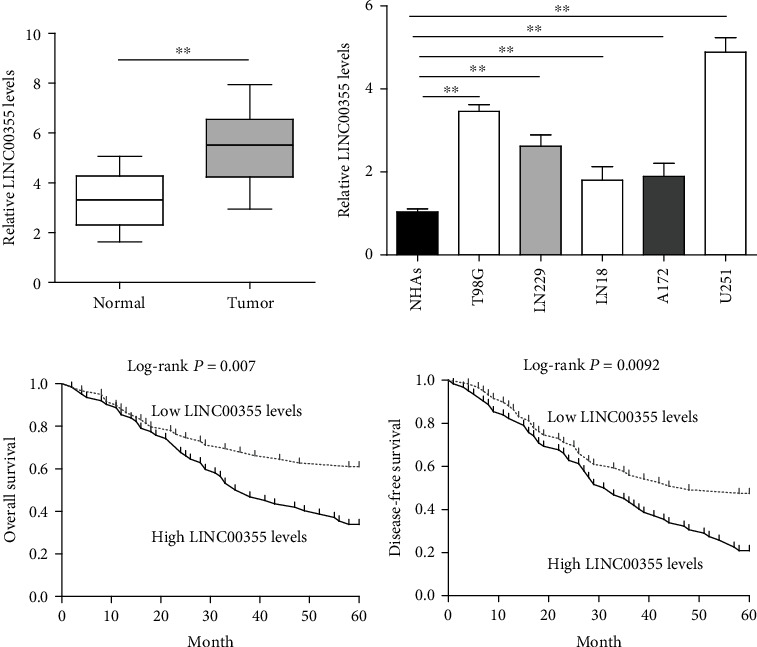
The dysregulation of LINC00355 in glioma and its clinical significance. (a) LINC00355 was frequently upregulated in glioma tissues compared with their matched nontumor specimens according to quantitative PCR. (b) The expression levels of LINC00355 in glioma cell lines and normal cells were tested by qRT-PCR. (c, d) The relationships between LINC00355 expression and overall survival and disease-free survival in glioma patients. ^∗^*p* < 0.05 and ^∗∗^*p* < 0.01.

We firstly performed RT-PCR to detect the levels of LINC00355 in 121 glioma patients from our hospital.  Figure 1(a) shows that LINC00355 expressions were distinctly upregulated in glioma specimens compared with matched normal tissues. In addition, five glioma cell lines were also observed to exhibit increased LINC00355 expression compared to normal NHAs (Figure 1(b)). Then, we further explored the clinical significance of LINC00355 in glioma patients. As shown in Table 2, high LINC00355 expression was associated with KPS (*p* = 0.019) and WHO grade (*p* = 0.024). Subsequent clinical research confirmed the prognostic value of LINC00355 whose upregulation was associated with shorter OS (*p* = 0.0023, Figure 1(c)) and DFS (*p* = 0.0092, Figure 1(d)). More importantly, multivariate Cox regression analysis confirmed miR-1225 as an independent indicator in prediction of the OS (HR = 2.785, 95% CI: 1.201-3.887, *p* = 0.015, Table 3) and DFS (HR = 2.657, 95% CI: 1.375-3.778, *p* = 0.021, Table 4) of glioma patients. Overall, our findings revealed that LINC00355 expression was distinctly increased in glioma and associated with advanced clinical progress.

### 3.2. Depression of LINC00355 Inhibited the Proliferation of Glioma Cells and Accelerated Cell Apoptosis

**Figure 2:**
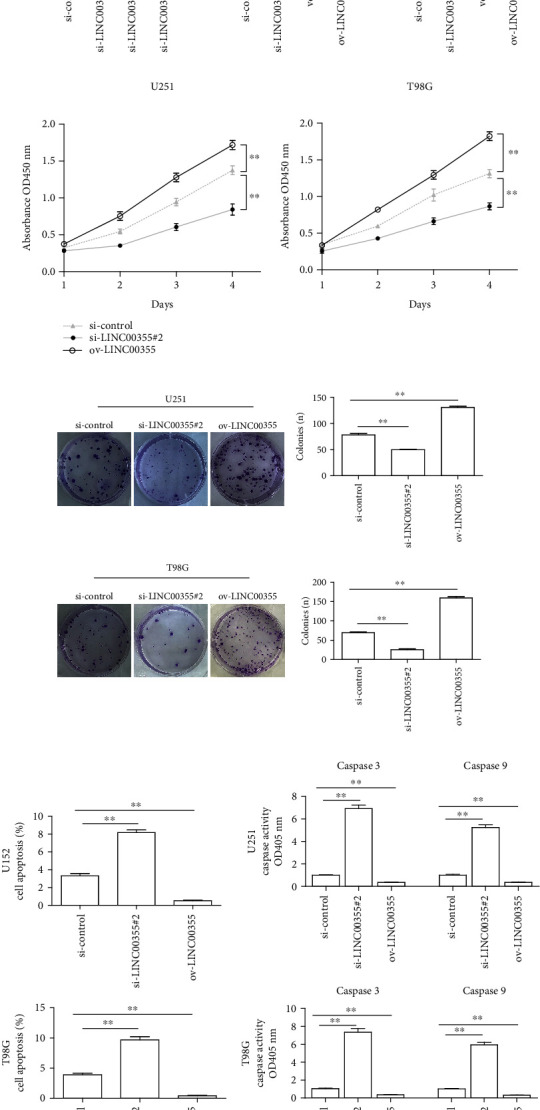
Changes in LINC00355 expression affected the proliferation and apoptosis of glioma cells. (a) qPCR detected LINC00355 levels in U251 cells after transfection with siRNAs against LINC00355 (si-LINC00355#1, si-LINC00355#2, and si-LINC00355#3). (b) The overexpressing efficiency by overexpressing plasmid (ov-LINC00355) transfection or knockdown efficiency by LINC00355#3 siRNAs was determined by qPCR analyses. (c) CCK-8 assays presented that the proliferative rates of U251 and T98G cells decreased after depletion of LINC00355, whereas the cell proliferation elevated after overexpressing LINC00355. (d) Clonogenic assays showed the clone number in glioma cells (magnification: 10x). (e) Flow cytometry showed the apoptosis of U251 and T98G cells after treatment. (f) The caspase 3/9 activities of glioma cells were examined. ^∗^*p* < 0.05 and ^∗∗^*p* < 0.01.

To assess the impacts of LINC00355 on the oncogenic behaviors of glioma cells, we first synthesized siRNAs against LINC00355 (si-LINC00355#1, si-LINC00355#2, and si-LINC00355#3) and subsequently transfected these LINC00355 siRNAs into U251 and T98G cells because LINC00355 expression was relatively higher in U251 and T98G cells than other glioma cells. Real-time PCR assays were then employed to evaluate the knockdown efficiency. The data suggested that si-LINC00355#2 had the highest knockdown efficiency (Figure 2(a)). Afterwards, qPCR was also conducted to determine the LINC00355 levels in glioma cells after transfection with LINC00355 overexpressing plasmids (ov-LINC00355) or si-LINC00355#2 (Figure 2(b)). Subsequently, to explore whether LINC00355 affected cellular growth, CCK-8 analyses were performed. The data revealed that repressing the expression of LINC00355 remarkably depressed the proliferative rates of glioma cells, while ectopic expression of LINC00355 led to notably increased growth curves (Figure 2(c)). Then, the influence of LINC00355 on cellular colony formation was also assessed. The data demonstrated that, when LINC00355 was knocked down, the number of colonies was obviously reduced, whereas the colony number was markedly increased in glioma cells when they were transfected with LINC00355 overexpressing plasmids (Figure 2(d)). Additionally, we also assayed the impact of LINC00355 on glioma cell apoptosis. Data from flow cytometry analyses confirmed that overexpressing LINC00355 significantly decreased the apoptosis rates, while depression of LINC00355 dramatically promoted the apoptosis of glioma cells (Figure 2(e)). Moreover, the caspase 3/9 activities were also evaluated and the data proved that enhancing expression of LINC00355 remarkably reduced the activities of caspase 3/9 in glioma cells, whereas depletion of LINC00355 significantly increased the caspase 3/9 activities (Figure 2(f)). Taken together, our results suggested that LINC00355 strongly promoted the development of glioma.

### 3.3. LINC00355 Depletion Repressed the Metastatic Potentials of Glioma Cells

**Figure 3:**
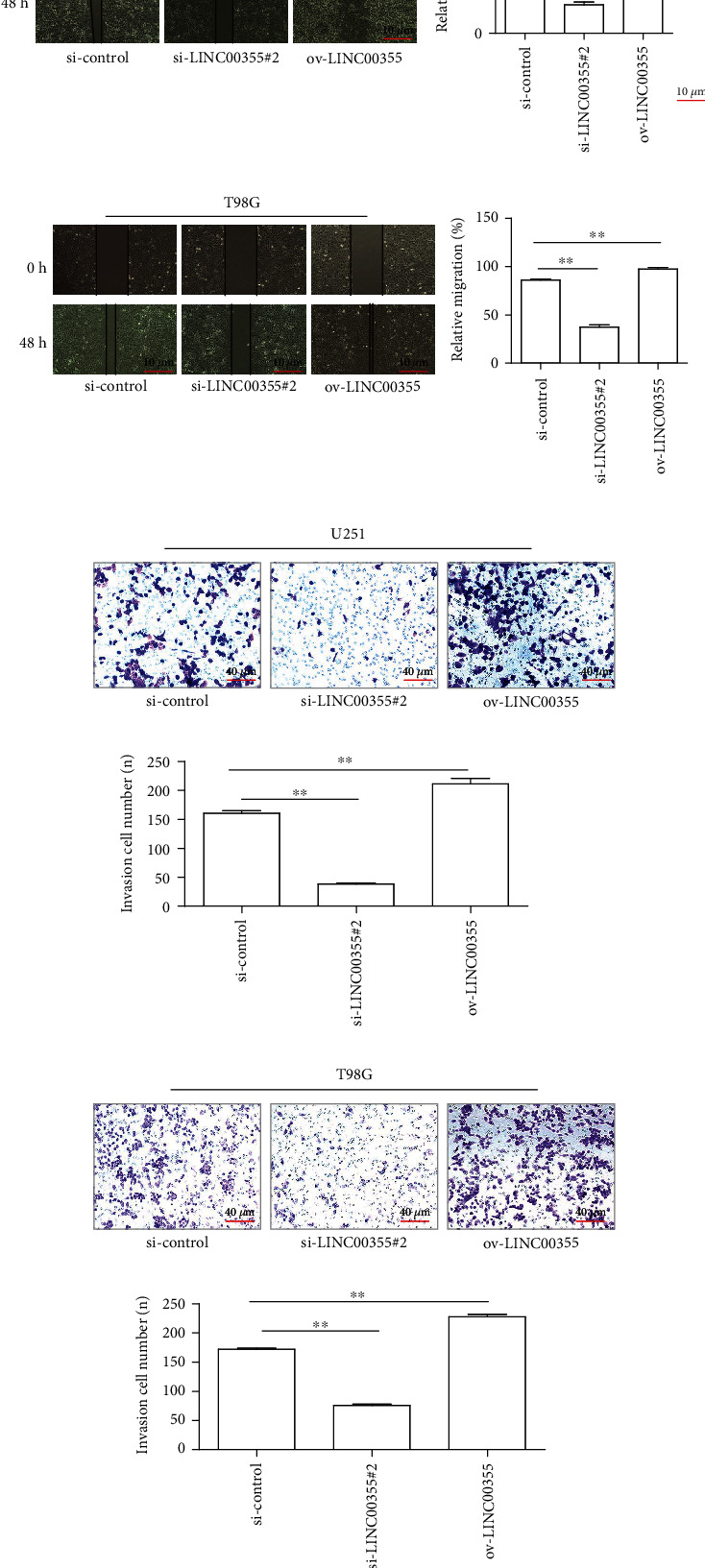
The impact of LINC00355 expressing changes on the metastasis of glioma cells. (a, b) The effects of LINC00355 on glioma cell migration were analyzed by wound healing assays (magnification: 10x). (c, d) Transwell assay showed the invaded cell number in U251 and T98G cells after treatment (magnification: 40x). ^∗^*p* < 0.05 and ^∗∗^*p* < 0.01.

Next, we further investigated the effects of LINC00355 on the cellular metastasis, a critical determinant of malignant progression. First, we employed wound healing analyses to determine the impact of LINC00355 on the mobility of glioma cells. The results indicated that the glioma cells treated with the LINC00355 siRNAs displayed a significant reduction in the migratory activity, whereas the glioma cells treated with ov-LINC00355 plasmids markedly enhanced cellular migration (Figures 3(a) and 3(b)). The Transwell assays were also applied. When the expression of LINC00355 was enhanced in glioma cells, the cell invasion ability was noticeably increased, while inhibition of LINC00355 obviously decreased the invasion capabilities of glioma cells (Figures 3(c) and 3(d)). Therefore, these results validated that LINC00355 enhanced the metastatic potentials of glioma cells.

### 3.4. miR-1225 Was a Target of LINC00355 in Glioma Cells

**Figure 4:**
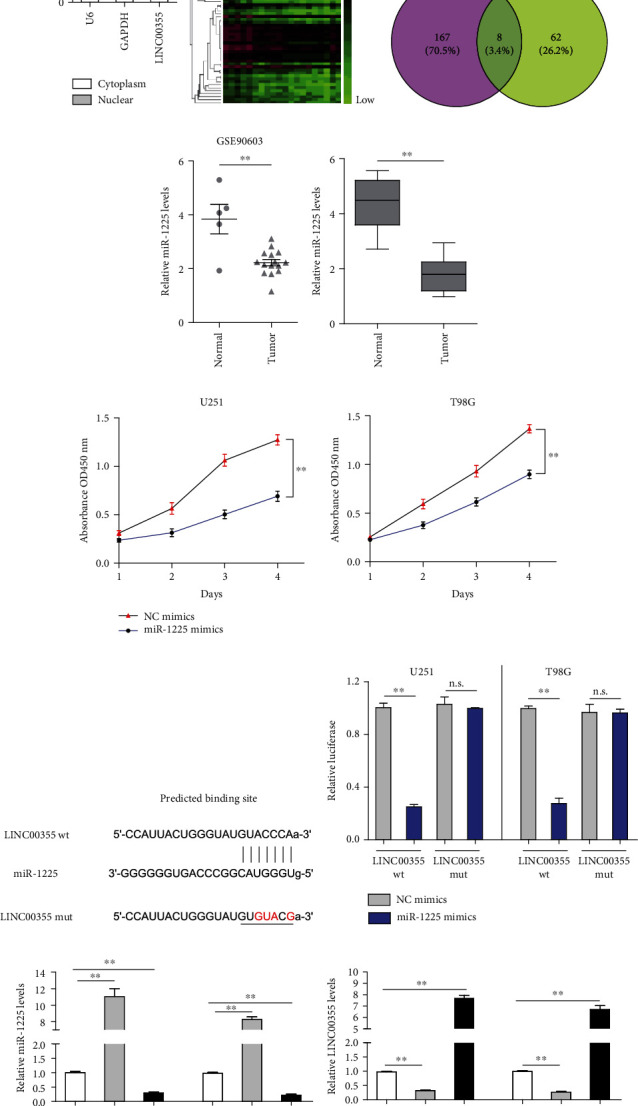
LINC00355 sponged miR-1225 in glioma cells. (a) Subcellular fractionation assay clarified the subcellular location of LINC00355 in U251 cells. (b) The heat map of differentially expressed miRNAs in GSE90603 data. (c) The intersection between the downregulated miRNAs in glioma tumor tissues and the predicting target miRNAs using miRDB program. (d) The relative expression of miR-1225 in GSE90603 data. (e) The expressions of miR-1225 in 121 glioma tumor specimens. (f) CCK-8 assays. (g) miRDB program predicted the binding sites between LINC00355 and miR-1225. (h) Luciferase activity detection. (i, j) qPCR examined the expressions of miR-1225 (i) and LINC00355 (j) in glioma cells after the transfection. ^∗^*p* < 0.05 and ^∗∗^*p* < 0.01.

Subsequently, the molecular mechanism was investigated. Since previous reports had demonstrated that lncRNAs which distributed in cytoplasm exerted their functions through sponging miRNAs, we thereby determined the subcellular localization of LINC00355 [17]. The data suggested that LINC00355 is mainly distributed in the cytoplasm (Figure 4(a)). Hence, our group delved into the downstream miRNAs of LINC00355. Bioinformatics analysis using GSE90603 was applied to obtain the differentially expressed miRNAs in glioma tumor tissues. The heat map is shown in Figure 4(b). We then obtained eight miRNAs by intersecting the downregulated miRNAs in glioma tumor tissues and predicting the target miRNAs using miRDB program [18] (Figure 4(c)). Among these eight miRNAs, miR-1225, a tumor suppressor previously reported, attracted our attention, and we attempted to explore whether it was a target of LINC00355 [19, 20]. The relative expression of miR-1225 in GSE90603 data is shown in Figure 4(d). Besides, qPCR assays revealed that miR-1225 expression was deceased in glioma specimens (Figure 4(e)). In addition, functional studies with CCK-8 assays validated that forced expression of miR-1225 resulted in remarkable suppression of glioma cellular growth (Figure 4(f)). Moreover, the binding site between LINC00355 and miR-1225 which was predicted by miRDB program is presented in Figure 4(g). Afterwards, luciferase reporter assays were employed to verify their interaction. As shown in Figure 4(h), miR-1225 overexpression suppressed the activities of LINC00355 wild-type (WT) reporters but not LINC00355 mutant-type (MUT) reporters, which confirmed their direct interaction. Furthermore, qPCR validated that LINC00355 knockdown led to elevated expression of miR-1225, whereas LINC00355 ectopic expression markedly inhibited miR-1225 levels (Figure 4(i)). Additionally, the data from qPCR also confirmed that enhancing miR-1225 expression contributed to the suppression of LINC00355, while miR-1225 deficiency resulted in notably increased expression of LINC00355 (Figure 4(j)). Collectively, the above findings suggested that LINC00355 sponged miR-1225 in glioma cells.

### 3.5. miR-1225 Targeted FNDC3B in Glioma Cells

**Figure 5:**
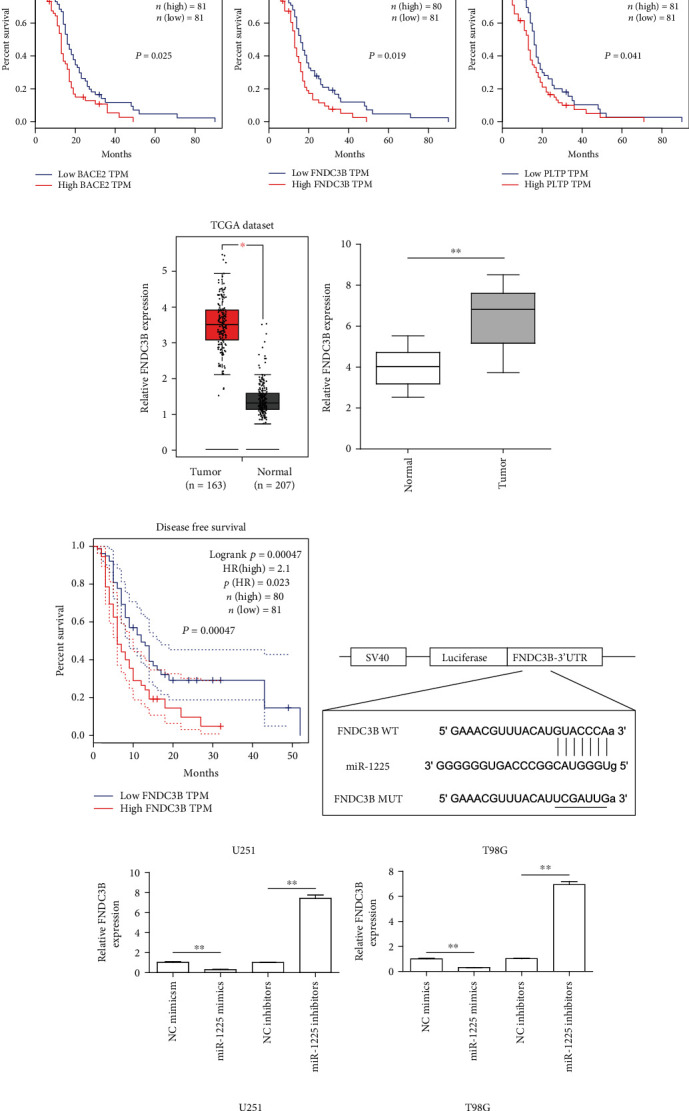
FNDC3B was a direct target of miR-1225 in glioma cells. (a) The intersection of the predicting results of miRDB and TargetScan, and 1000 upregulated genes in glioma tumor samples. (b) GEPIA program analyzed the overall survivals of BACE2, FNDC3B, and PLTP in glioma using TCGA data. (c) GEPIA algorithm analyzed the FNDC3B levels in glioma tissues and matched normal tissues using TCGA dataset. (d) qPCR determined the levels of FNDC3B in 121 glioma tumor samples. (e) GEPIA program analyzed the disease-free survival of FNDC3B in GBM. (f) Binding sequences between miR-1225 and FNDC3B were predicted using miRDB algorithm. (g) RT-PCR was performed for the examination of the levels of FNDC3B in glioma cells after miR-1225 was dysregulated. (h) Luciferase reporter assay confirmed the molecular binding. ^∗^*p* < 0.05 and ^∗∗^*p* < 0.01.

In order to further elucidate how miR-1225 contributed to glioma growth and metastasis, bioinformatics algorithms, miRDB and TargetScan, were then employed to identify the target genes that mediated the effects of miR-1225 on glioma. We then intersected the predicting results of TargetScan, miRDB, and 1000 upregulated genes in tumor samples (using TCGA data analyzed by GEPIA program [21]). We found 14 common genes (Figure 5(a)). The OS of these 14 genes in glioma were subsequently analyzed by the GEPIA program, and we found that only BACE2, FNDC3B, and PLTP were significantly correlated with OS (Figure 5(b)). Among the three genes, FNDC3B attracted our attention because of its association with tumorigenesis and metastasis of various cancer types [22, 23]. Data from TCGA datasets revealed that FNDC3B levels were increased in glioma specimens (Figure 5(c)). Moreover, qPCR also confirmed that FNDC3B was upregulated in 121 glioma samples (Figure 5(d)). Furthermore, bioinformatics analyses using GEPIA program also indicated that patients with high FNDC3B had significantly lower disease-free survivals (Figure 5(e)). The predicting binding site between miR-1225 and 3′-UTR of FNDC3B mRNA is presented in Figure 5(f). Besides, the FNDC3B levels in glioma cells were notably depressed when the cells were transfected with miR-1225 mimics, while miR-1225 inhibitors remarkably promoted the FNDC3B levels in glioma cells, which indicated that miR-1225 expressions were negatively correlated with FNDC3B expressions (Figure 5(g)). Hence, we next performed luciferase reporter assays to certify whether FNDC3B was a direct target gene of miR-1225. The data suggested that overexpression of miR-1225 remarkably repressed the luciferase activity of glioma cells transfected with wild-type (WT) predicted binding site luciferase reporter vectors, while miR-1225 had no influence on the luciferase activity in cells transfected with mutant (MUT) predicted binding site luciferase reporter vectors (Figure 5(h)). Therefore, these data proved that FNDC3B was the exact target of miR-1225 in glioma cells.

### 3.6. LINC00355 Modulated FNDC3B via miR-1225 in Glioma Cells

**Figure 6:**
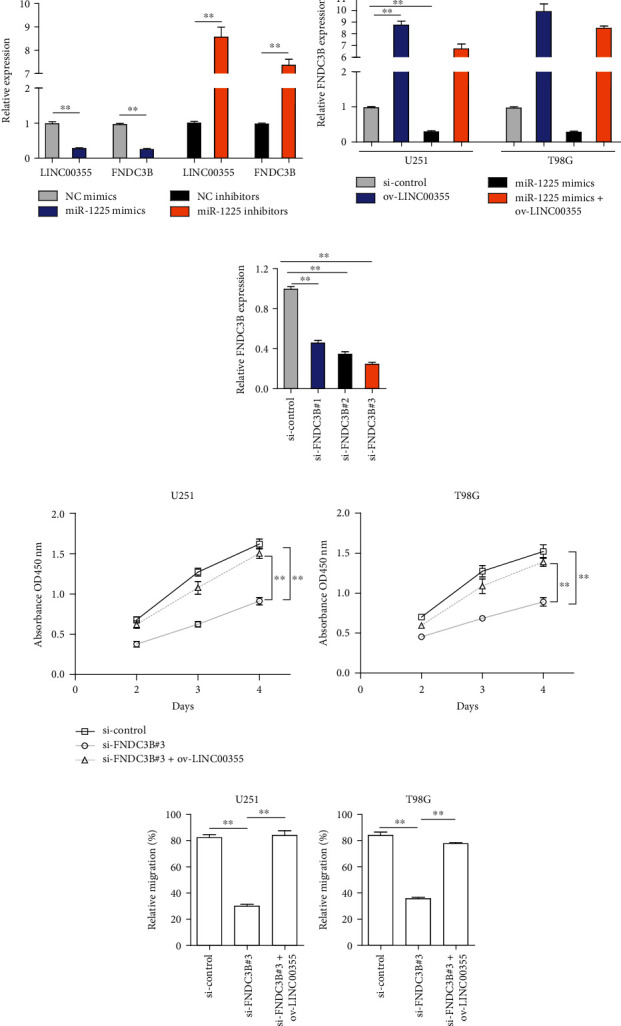
LINC00355 modulated glioma malignant behaviors via miR-1225/FNDC3B axis. (a) RT-PCR determined the expressions of LINC00355 and FNDC3B in glioma cells transfected with miR-1225 mimics or inhibitors. (b) The expressions of FNDC3B in glioma cells after various treatments by RT-PCR. (c) The levels of FNDC3B in U251 cells after treatment with FNDC3B siRNAs (si-FNDC3B#1, si-FNDC3B#2, and si-FNDC3B#3). (d) CCK-8 assays. (e) Relative migration of glioma cells was evaluated by wound healing assays. ^∗^*p* < 0.05 and ^∗∗^*p* < 0.01.

Since we had demonstrated that LINC00355 sponged miR-1225, and miR-1225 targeted FNDC3B in glioma cells, we wondered whether LINC00355 could regulate FNDC3B. Firstly, qPCR was utilized for determining the levels of LINC00355 and FNDC3B in glioma cells after their miR-1225 was depleted or overexpressed. It was observed that enhancing expressions of miR-1225 caused remarkably decreased levels of LINC00355 and FNDC3B, while knocking down miR-1225 was able to elevate LINC00355 and FNDC3B expression (Figure 6(a)). Then, ectopic expression of LINC00355 was capable to promote FNDC3B expression, and LINC00355 overexpression could also abrogate the suppressor functions of miR-1225 on FNDC3B expression (Figure 6(b)). Subsequently, our group synthesized siRNAs targeting FNDC3B (si-FNDC3B#1, si-FNDC3B#2, and si-FNDC3B#3) and applied qPCR to detect the knockdown efficiency (Figure 6(c)). The data suggested that si-FNDC3B#3 had the highest knockdown efficiency. Then, we performed functional experiments to investigate whether LINC00355 could restore the inhibitory influence of FNDC3B siRNAs on cellular growth and migration. CCK-8 assays demonstrated that depression of FNDC3B by si-FNDC3B#3 inhibited the cell proliferation of glioma cells, while reintroduction of LINC00355 was able to reverse the cell proliferation which was suppressed by si-FNDC3B#3 (Figure 6(d)). Similar results from wound healing assays were also observed that LINC00355 overexpression could restore the suppressive impacts of si-FNDC3B#3 on glioma cell migration (Figure 6(e)). Taken together, the above data indicated that LINC00355 modulated glioma malignant behaviors via miR-1225/FNDC3B axis.

## 4. Discussion

Human gliomas are the most common tumors which frequently occur in the central nervous system. The poor clinical prognosis of patients suffering from this malignancy resulted in the large-scale investigation of sensitive cancer biomarkers due to their critical effects on the clinical management of patients [24, 25]. Although more and more tumor-related genes, gene methylation, transcription factors, and other molecules were identified to have potential as biomarkers, the genuine clinical application of these possible biomarkers is limited [26]. In recent years, more and more researches highlighted the biological significance of lncRNAs in tumor progression and emerging evidences indicated the important diagnostic and prognostic values of lncRNAs in various tumor patients [27, 28]. Here, a new glioma-associated lncRNA, LINC00355, which was upregulated in glioma and predicted advanced clinical progress, was identified. Besides, by analyzing five-year survival data, we confirmed that upregulation of LINC00355 was associated with shorter OS and DFS of glioma patients and could be an independent poor prognostic factor using multivariate Cox model. Overall, our findings provided the first evidence that LINC00355 could be used as a reliable biomarker for glioma patients.

Previously, the frequent dysregulation of LINC00355 has been demonstrated in several cancers, such as bladder cancer, colorectal tumor, and lung cancer [29–31]. However, the function of LINC00355 in tumor progression was rarely reported. Lu et al. [30] showed that LINC00355 acted as a positive regulator in cell growth and metastasis of head and neck squamous cell carcinoma cells via acting as a miRNA-195 sponge to increase the levels of HOXA10. Liang et al. [29] showed that LINC00355 was overexpressed in lung cancer and its forced expression resulted in promoted proliferation via modulating miRNA-195/CCNE1. In this research, our group observed that its knockdown suppressed the proliferation, migration, and invasion of glioma cells. In addition, in our gain-of-function assays, we observed that overexpression of LINC00355 displayed an opposite result compared to the downregulation of LINC00355 in glioma cells. Our findings firstly confirmed LINC00355 as a tumor promoter in glioma, which was in line with the tumor-promotive roles of LINC00355 in lung cancer and head and neck squamous cell carcinoma.

It has been demonstrated that lncRNAs acted as sponges for miRNAs and abrogated the inhibitive effects of miRNAs on the targeted transcripts [17, 32]. Previously, LINC00355 acting as a molecular sponge of miRNAs was reported in the above two tumors [29, 30]. To further explore the potential mechanism involved in ceRNA hypothesis, our group firstly identified its localization in glioma cells because cytosolic lncRNAs have been demonstrated to modulate mRNA stability and act as miRNA sponge. Using bioinformatics analysis, we identified 8 miRNAs which may interact with LINC00355. Among these candidates, miR-1225 attracted our attention due to its distinct overexpression in glioma specimens and its forced expression suppressed the proliferation of U251 and T98G cells using CCK-8 assays. The data of RT-PCR revealed that miR-1225 expression was distinctly increased upon LINC00355 suppression. On the contrary, overexpression of miR-1225 reduced expressions of LINC00355, whereas suppression of miR-1225 exhibited a converse trend. Our findings revealed a converse correlation between LINC00355 and miR-1225. Besides, luciferase activity assays confirmed the direct binding relationship between LINC00355 and miR-1225. Previously, the distinct downregulation of miR-1225 and its role in acting as a tumor suppressor in several tumors have been reported [33, 34]. In glioma, Li et al. [19] reported that miR-1225 was lowly expressed in glioblastoma and its overexpression suppressed the proliferation and metastasis of glioblastoma cells through targeting IRS1. Thus, our findings, together with previous studies, indicated that LINC00355 exhibited its tumor-promotive roles via the inhibition of miR-1225.

Emerging evidences indicated that suppression of FNDC3B was involved in the inhibition of the ability of cell growth, adhesion, and invasion, suggesting that FNDC3B may display functional effects on cell's progress [35]. Thus, whether FNDC3B acted as a functional regulator attracted increasing attention. In recent years, overexpression of FNDC3B and its tumor-promotive roles have been reported in several tumors, such as hepatocellular carcinoma, tongue squamous cell carcinoma, and glioma [22, 23, 36]. Importantly, FNDC3B played an important role in the progress of the EMT pathway. In addition, it was reported that several miRNAs exhibited their function by targeting FNDC3B. For instance, Fan et al. [37] reported that overexpression of miR-143 promoted prostate cancer cell migration and invasion via the regulation of FNDC3B. Xu et al. [22] showed that miR-129-5p acted as a tumor suppressor in glioblastoma because its upregulation suppressed cell proliferation and metastasis via targeting FNDC3B. However, there is limited evidence about the exact molecular mechanism involved in FNDC3B-mediated glioma progression. In this study, we analyzed TCGA datasets to study the clinical significance of FNDC3B in glioma patients, finding that higher levels of FNDC3B predicted poorer clinical prognosis. Then, performing bioinformatics analysis, it was confirmed that FNDC3B may be a potential target of miR-1225. Moreover, using the luciferase assays, we confirmed the fact that the luciferase activity could be distinctly reduced by forced overexpression of miR-1225. Moreover, we performed a series of cell experiments to study the association between miR-1225 and FNDC3B, finding that further overexpression of FNDC3B in glioma cells transfected in miR-1225 mimics could reverse the inhibitory effects of miR-1225 mimics on glioma cell migration and proliferation, indicating that miR-1225 played a critical role in carcinogenesis via inhibition of FNDC3B. Interestingly, we found that LINC00355 increased FNDC3B expression through suppressing miR-1225. Further functional assays revealed that restoration of LINC00355 rescued the effect of the FNDC3B knockdown on cell growth and migration. These data revealed that LINC00355 exhibited its oncogenic effects at least in part via the regulation of miR-1225/FNDC3B axis.

Our study had several limitations: firstly, the small sample size of the present study was a limitation. Secondly, the potential mechanisms involved in LINC00355 overexpression in glioma remained unknown. Thirdly, the downstream molecules which FNDC3B regulated were not studied.

## 5. Conclusion

Collectively, we firstly indicated that LINC00355 expression was distinctly upregulated in glioma specimens and cells and its increased expressions may be a new biomarker for diagnosis and prognosis for glioma patients. The oncogenic functions of LINC00355 may achieve partially through the modulation of miR-1225/FNDC3B axis. Overall, LINC00355/miR-1225/FNDC3B network may become a candidate target for glioma therapy.

## Figures and Tables

**Table 1:** Primers used for RT-PCR.

Names	Sequences (5′-3′)
LINC00355-F	GTTGGTGCCTGCTTTTCCAC
LINC00355-R	GGCGTGATACAACTGTCTGC
miR-1225-F	CTATGCTCTAGGATCCTCCTG
miR-1225-R	GCAAATCAGAATCACACCTG
FNDC3B-F	CCCTCCCTATCTGACTCACCA
FNDC3B-R	GGTCCGGTAACAGGTGGGTA
GAPDH-F	GCCCAATACGACCAAATCC
GAPDH-R	AGCCACATCGCTCAGACAC
U6-F	GTGCTCGCTTCGGCAGC
U6-R	CCAGTGCAGGGTCCGAGGT

**Table 2:** The association between LINC00355 expression and clinicopathological factors in glioma patients.

Parameters	Number	LINC00355 expression		*p*
		High	Low	
Age				0.512
<50	52	24	28	
≥50	69	36	33	
Gender				0.409
Male	64	34	30	
Female	57	26	31	
Tumor location				0.238
Supratentorial	60	33	27	
Infratentorial	61	27	34	
Family history of cancer				0.410
Yes	44	24	20	
No	77	36	41	
KPS				0.019
≥70	74	43	31	
<70	47	17	30	
Size				0.158
<5 cm	73	40	33	
≥5 cm	48	20	28	
WHO grade				0.024
Low	81	46	35	
High	40	14	26	

**Table 3:** Univariate and multivariate analyses of overall survival in 121 patients with glioma.

Factors	Univariate analysis		Multivariate analysis	
	*p* value	HR (95% CI)	*p* value	HR (95% CI)
Age	0.377	1.482 (0.782-2.446)	—	—
Gender	0.259	1.762 (0.892-2.736)	—	—
Tumor location	0.336	1.528 (0.631-2.147)	—	—
Family history of cancer	0.175	1.499 (0.892-2.428)	—	—
KPS	0.008	3.182 (1.428-4.667)	0.016	2.576 (1.283-4.156)
Size	0.092	1.889 (1.028-2.889)	—	—
WHO grade	0.001	3.562 (1.382-5.012)	0.006	2.984 (1.148-4.556)
LINC00355 expression	0.004	3.018 (1.582-4.118)	0.015	2.785 (1.201-3.887)

**Table 4:** Univariate and multivariate analyses of disease-free survival in 121 patients with glioma.

Factors	Univariate analysis		Multivariate analysis	
	*p* value	HR (95% CI)	*p* value	HR (95% CI)
Age	0.261	0.732 (0.385-1.662)	—	—
Gender	0.385	0.829 (0.448-2.185)	—	—
Tumor location	0.582	1.128 (0.626-1.896)	—	—
Family history of cancer	0.227	1.377 (0.872-2.155)	—	—
KPS	0.005	3.258 (1.385-4.992)	0.009	3.019 (1.184-4.462)
Size	0.148	0.998 (0.682-2.472)	—	—
WHO grade	0.001	3.662 (1.427-5.372)	0.004	3.261 (1.285-4.665)
LINC00355 expression	0.008	2.899 (1.258-4.565)	0.021	2.657 (1.375-3.778)

## Data Availability

The data used to support the findings of the present study are available from the corresponding author upon reasonable request.

## References

[1] Siegel R. L., Miller K. D., Jemal A. (2018). Cancer statistics, 2018. *CA: a Cancer Journal for Clinicians*.

[2] Omuro A., DeAngelis L. M. (2013). Glioblastoma and other malignant gliomas: a clinical review. *JAMA*.

[3] Agnihotri S., Burrell K. E., Wolf A. (2013). Glioblastoma, a brief review of history, molecular genetics, animal models and novel therapeutic strategies. *Archivum Immunologiae et Therapiae Experimentalis (Warsz)*.

[4] Bush N. A., Chang S. M., Berger M. S. (2017). Current and future strategies for treatment of glioma. *Neurosurgical Review*.

[5] Matsui M., Corey D. R. (2017). Non-coding RNAs as drug targets. *Nature Reviews. Drug Discovery*.

[6] Andersen R. E., Lim D. A. (2018). Forging our understanding of lncRNAs in the brain. *Cell and Tissue Research*.

[7] Jarroux J., Morillon A., Pinskaya M. (2017). History, discovery, and classification of lncRNAs. *Advances in Experimental Medicine and Biology*.

[8] Ma L., Bajic V. B., Zhang Z. (2013). On the classification of long non-coding RNAs. *RNA Biology*.

[9] Akhade V. S., Pal D., Kanduri C. (2017). Long Noncoding RNA: Genome organization and mechanism of action. *Advances in Experimental Medicine and Biology*.

[10] Marchese F. P., Raimondi I., Huarte M. (2017). The multidimensional mechanisms of long noncoding RNA function. *Genome Biology*.

[11] Peng W. X., Koirala P., Mo Y. Y. (2017). lncRNA-mediated regulation of cell signaling in cancer. *Oncogene*.

[12] Martens-Uzunova E. S., Bottcher R., Croce C. M., Jenster G., Visakorpi T., Calin G. A. (2014). Long noncoding RNA in prostate, bladder, and kidney cancer. *European Urology*.

[13] Wu Y., Wang P. S., Wang B. G. (2018). Genomewide identification of a novel six-lncRNA signature to improve prognosis prediction in resectable hepatocellular carcinoma. *Cancer Medicine*.

[14] Gawronski A. R., Uhl M., Zhang Y. (2018). MechRNA: prediction of lncRNA mechanisms from RNA-RNA and RNA-protein interactions. *Bioinformatics*.

[15] Vishnoi A., Rani S. (2017). miRNA biogenesis and regulation of diseases: an overview. *Methods in Molecular Biology*.

[16] Di Leva G., Garofalo M., Croce C. M. (2014). MicroRNAs in cancer. *Annual Review of Pathology*.

[17] Salmena L., Poliseno L., Tay Y., Kats L., Pandolfi P. P. (2011). A *ceRNA* Hypothesis: The Rosetta Stone of a Hidden RNA Language?. *Cell*.

[18] Chen Y., Wang X. (2020). miRDB: an online database for prediction of functional microRNA targets. *Nucleic Acids Research*.

[19] Li D., Chi G., Chen Z., Jin X. (2018). MicroRNA-1225-5p behaves as a tumor suppressor in human glioblastoma via targeting of IRS1. *Oncotargets and Therapy*.

[20] Sun P., Zhang D., Huang H. (2019). MicroRNA-1225-5p acts as a tumor-suppressor in laryngeal cancer via targeting CDC14B. *Biological Chemistry*.

[21] Tang Z., Li C., Kang B., Gao G., Li C., Zhang Z. (2017). GEPIA: a web server for cancer and normal gene expression profiling and interactive analyses. *Nucleic Acids Research*.

[22] Xu H., Hu Y., Qiu W. (2017). Potential mechanisms of microRNA-129-5p in inhibiting cell processes including viability, proliferation, migration and invasiveness of glioblastoma cells U87 through targeting _FNDC3B_. *Biomedicine & Pharmacotherapy*.

[23] Zhong Z., Zhang H., Hong M. (2018). FNDC3B promotes epithelial-mesenchymal transition in tongue squamous cell carcinoma cells in a hypoxic microenvironment. *Oncology Reports*.

[24] Gusyatiner O., Hegi M. E. (2018). Glioma epigenetics: from subclassification to novel treatment options. *Seminars in Cancer Biology*.

[25] Ludwig K., Kornblum H. I. (2017). Molecular markers in glioma. *Journal of Neuro-Oncology*.

[26] Davis M. E. (2018). Epidemiology and overview of gliomas. *Seminars in Oncology Nursing*.

[27] Chandra Gupta S., Nandan T. Y. (2017). Potential of long non-coding RNAs in cancer patients: from biomarkers to therapeutic targets. *International Journal of Cancer*.

[28] Schmitt A. M., Chang H. Y. (2016). Long noncoding RNAs in cancer pathways. *Cancer Cell*.

[29] Liang Y., Rong X., Luo Y. (2020). A novel long non-coding RNA LINC00355 promotes proliferation of lung adenocarcinoma cells by down-regulating miR-195 and up-regulating the expression of CCNE1. *Cellular Signalling*.

[30] Lu S., Sun Z., Tang L., Chen L. (2020). LINC00355 promotes tumor progression in HNSCC by hindering microRNA-195-mediated suppression of HOXA10 expression. *Molecular Therapy - Nucleic Acids*.

[31] Seitz A. K., Christensen L. L., Christensen E. (2017). Profiling of long non-coding RNAs identifies LINC00958 and LINC01296 as candidate oncogenes in bladder cancer. *Scientific Reports*.

[32] Fu C., Li D., Zhang X., Liu N., Chi G., Jin X. (2018). lncRNA PVT1 facilitates tumorigenesis and progression of glioma via regulation of miR-128-3p/GREM1 axis and BMP signaling pathway. *Neurotherapeutics*.

[33] Stang A., Weilert H., Lipp M. J. (2021). MicroRNAs in blood act as biomarkers of colorectal cancer and indicate potential therapeutic targets. *Molecular Oncology*.

[34] Gao S., Shi P., Tian Z., Yang X., Liu N. (2021). Overexpression of miR-1225 promotes the progression of breast cancer, resulting in poor prognosis. *Clinical and Experimental Medicine*.

[35] Cai C., Rajaram M., Zhou X. (2012). Activation of multiple cancer pathways and tumor maintenance function of the 3q amplified oncogene FNDC3B. *Cell Cycle*.

[36] Lin C. H., Lin Y. W., Chen Y. C. (2016). FNDC3B promotes cell migration and tumor metastasis in hepatocellular carcinoma. *Oncotarget*.

[37] Fan X., Chen X., Deng W., Zhong G., Cai Q., Lin T. (2013). Up-regulated microRNA-143 in cancer stem cells differentiation promotes prostate cancer cells metastasis by modulating FNDC3B expression. *BMC Cancer*.

